# Resilience is associated with frailty and older age in hospitalised patients

**DOI:** 10.1186/s12877-022-03251-9

**Published:** 2022-07-10

**Authors:** Marco Vincenzo Lenti, Alice Silvia Brera, Alessia Ballesio, Gabriele Croce, Lucia Padovini, Giampiera Bertolino, Antonio Di Sabatino, Catherine Klersy, Gino Roberto Corazza

**Affiliations:** 1 Department of Internal Medicine, University of Pavia, Pavia, Italy; 2 Clinical Epidemiology & Biometry, Fondazione IRCCS Policlinico San Matteo, University of Pavia, Pavia, Italy; 3 Department of Internal Medicine, Clinica Medica, Fondazione IRCCS Policlinico San Matteo, Viale Golgi 19, 27100 Pavia, Italy

**Keywords:** Ageing, Clinical complexity, Elderly, Multimorbidity

## Abstract

**Background:**

Little is known about resilience in an internal medicine setting. We aimed to assess the relationship between resilience and frailty and other clinical and sociodemographic characteristics in a cohort of prospectively enrolled hospitalised patients.

**Methods:**

In 2017–2019, we consecutively enrolled patients in our internal medicine wards. We selected all patients who filled in the 25-item Connor-Davidson resilience scale (CD-RISC). Mean resilience was evaluated according to baseline demographic (i.e., age, sex, marital and socioeconomic status) and clinical (i.e., Cumulative Illness Rating Scale [CIRS], Edmonton Frail Scale [EFS], Barthel index, Short Blessed test, length of stay [LOS]) data. A multivariable analysis for assessing factors affecting resilience was fitted.

**Results:**

Overall, 143 patients (median age 69 years, interquartile range 52–79, 74 females) were included. Resilience was significantly lower in frail (*p* = 0.010), elderly (*p* = 0.021), dependent (*p* = 0.032), and more clinically (*p* = 0.028) and cognitively compromised patients (*p* = 0.028), and in those with a low educational status (*p* = 0.032). No relation between resilience and LOS was noticed (*p* = 0.597). Frail patients were significantly older (*p* < 0.001), had a greater disease burden as measured by CIRS comorbidity (*p* < 0.001) and severity indexes (*p* < 0.001), were more dependent (*p* < 0.001), more cognitively impaired (*p* < 0.001), and displayed a lower educational level (*p* = 0.011) compared to non-frail patients. At multivariable analysis, frailty (*p* = 0.022) and dependency (*p* = 0.031; according to the Barthel index) were associated with lower resilience in the age groups 18–64 and ≥ 65 years, respectively.

**Conclusions:**

Low resilience was associated with frailty and dependency with an age-dependent fashion. Studies assessing the impact of this finding on important health outcomes are needed.

**Trial registration:**

Clinical Complexity in Internal Medicine Wards. San MAtteo Complexity Study (SMAC); NCT03439410. Registered 01/11/2017.

**Supplementary Information:**

The online version contains supplementary material available at 10.1186/s12877-022-03251-9.

## Background

Resilience is a key variable that is rising an ever-great interest among clinicians [1, 2] and consists of a process of negotiation, adaptation or management of stressors, encompassing a broad spectrum of personal abilities that allow bouncing back despite adversities [1, 3]. At present, most of the available data about resilience derive from disease-specific settings, such as psychiatric disorders, HIV, neoplastic diseases, and intestinal diseases [4–10], whereas little is known about resilience on a patient-centred basis, particularly in a hospital setting. Given its nature, resilience is multifactorial, and it has been shown to be affected by several factors, both patient- and environmental-related [1]. Remarkably, it can be enhanced through specific interventions, such as cognitive-behavioural therapy [11], and an increased resilience was found to be a predictor of recovery and quality of life in individuals living with chronic conditions [12–14].

Another factor that may theoretically affect resilience is frailty, which develops as a consequence of age-related decline in physiological systems, underlying a vulnerable state of health due to poor homeostatic resources [15, 16]. Indeed, frailty is associated with an increased risk of hospitalisation and mortality [17], and it can be reversed only at an early stage [18] through physical activity and nutritional interventions [19], as well as by using drugs targeting fundamental pathways of senescence [20]. Additionally, extra-biological factors, such as low family income, low educational level, and belonging to ethnic minorities, may represent, besides older age, additional contributors to this condition [21].

In clinical settings, both resilience and frailty can be assessed by means of internationally validated tools, such as the 25-item Connor-Davidson resilience scale (CD-RISC) [3] and the Edmonton Frail Scale (EFS) [22], but, disappointingly, their bidirectional relationship has never been studied. Hence, we aimed to assess the relationship between resilience and frailty and other clinical and sociodemographic characteristics in a cohort of prospectively enrolled hospitalised patients.

## Methods

The present research is part of the still ongoing San MAtteo Complexity (SMAC) study (NCT03439410), which is a prospective cohort study with the aim of validating a clinical complexity index, the general frame of which has already been described elsewhere [23, 24]. In brief, all adult patients (age > 18 years) admitted to our internal medicine ward, regardless of the cause, were consecutively enrolled from November 2017 to November 2019 according to the study protocol and are still currently being followed-up through a phone call. The San Matteo hospital Foundation is a tertiary referral, academic hospital located in Northern Italy, serving a population of roughly 500.000 people, and has a 24-h A&E access. Most patients (> 95%) who are admitted to our internal medicine ward have been first assessed by the emergency department that is in charge of sorting the admission to the various specialty wards.

Patients with a prognosis < 48 h and denial of informed consent were the only exclusion criteria. In case of advanced dementia, the informed consent was requested to the closest relative, caregiver or next of kin. We initially excluded those patients admitted with a severe or ominous condition (due to either the severity of a specific disease or the overall compromised clinical picture), with an expected prognosis of less than 48 h, according to the treating physician. If this was not the case, we eventually included these patients, after the 48-h observation. All the research staff involved into the project were specifically trained for collecting all data within the study [25].

For the specific purposes of the present study resilience was assessed in all consecutive patients, regardless of the admitting diagnosis, since 7^th^ January 2019 onwards, with the CD-RISC, a self-reported questionnaire that was translated and validated into Italian [3, 26]. The CD-RISC comprises of 25 items, each rated on a 5-point scale (0–4), with higher scores reflecting greater resilience [3]. According to the original scale [3], five areas are explored, namely personal competence, high standards, and tenacity (a); trust in one's instincts, tolerance of negative affect, and strengthening effects of stress (b); positive acceptance of change and secure relationships (c); control (d); and spiritual influences (e). Only patients who were unable (i.e., due to severe dementia, altered state of consciousness, linguistic barrier) or unwilling to complete the questionnaire, were excluded. A total of 143 patients (median age 69, IQR 52–79, 74 females), whose characteristics will be described later, were eventually included.

Frailty, according to the main study protocol, was assessed in the same 143 patients using a validated, and broadly used scale [22] that was translated into Italian. The EFS was initially designed as an easily available questionnaire to measure frailty in the elderly, both in a hospital and in an outpatient setting [22]. It consists of nine items that investigate cognition, health status, functional dependence, social support, medication use, nutrition, mood, continence, and functional performance. The score ranges from 0 to 2 per each item, with higher scores reflecting greater frailty [27]. A score of > 5 defines a frail state, and accordingly, we used this cut off for defining a patient as being frail.

All the causes of admission were collected according to the International Classification of Diseases (ICD)—9^th^ revision. Categories with less than 10 cases were pooled into the category “others”. Additionally, the overall burden of medical and psychiatric conditions was calculated by using the Cumulative Illness Rating Scale (CIRS) comorbidity and severity indexes [28]. While the CIRS comorbidity index reflects the burden of the number of diseases within the same patient, the severity index reflects how severe are the diseases on a 5-point scale (from 1, absence of the disease, to 5, life-threatening on a short term). Both indexes are calculated through a standardised algorithm [28].

Other relevant demographic or clinical data, as reported later, were retrieved from the dedicated REDCap database of the SMAC study, in which patients’ data were entered in a pseudo-anonymised format.

The primary aim of the study was to assess whether any relationship between resilience and frailty existed. As a secondary outcome, we assessed whether this relationship depended on a set of variables chosen a priori by the clinicians according to their relevance, namely age, sex, CIRS indexes, BMI, Barthel index (which assesses functional dependency) [29], income (categorised according to the limit of 1000€/month net salary below which people can be eligible for public subsidies in Italy), living alone, schooling (categorised into ≤ 8 or > 8 years of education, which reflects the compulsory education in Italy), the Short Blessed Test (which assesses cognitive impairment) [30], and length of stay (LOS). For the Barthel index, a cut off of < 60 was used for indicating moderate-to-severe dependency, as previously reported [31], while a cut off of ≥ 9 was used for the Short Blessed Test for indicating any grade of cognitive impairment, as originally reported in the validation study [30]. All the scales were assessed by the study investigators for each patient. Of the scales used in this research, only the CD-RISC is under license. The license was obtained from the scale owner (Prof. Davidson), by paying a fee for its use in an academic setting, in up to 1000 individuals.

All patients provided written informed consent prior to study enrolment and the study protocol was approved by the local Ethics Committee (IRCCS Policlinico San Matteo, 3 July 2017, Protocol number 2017/0019414).

### Statistical analysis

Continuous data were described with the mean and standard deviation or the median and interquartile range (IQR) and compared between EFS groups (≤ 5/ > 5) with the Student t test or the Mann Whitney U test, respectively, depending on the distribution. The distribution of continuous variables was assessed graphically by plotting the quantiles of the variable against the quantiles of normal distribution. Categorical data were reported as counts and percent and compared with the Fisher exact test. Univariable generalised linear models were then fitted for resilience, where the choice of covariates was decided a priori based on clinical considerations. Candidates were the most relevant patient clinical characteristics and the socioeconomic status. Variables with a *p*-value < 0.10 at the univariable model were included in the multivariable models. These were fitted in two predefined subgroups: adult patients (aged 18–64 years) and elderly patients (aged ≥ 65 years), according to the current WHO classification for developed countries. Spearman's rho was used for assessing the relation between resilience (continuous variable) and LOS. The software Stata 16 (StataCorp, College Station, TX, USA) was used for all computations. The study follows the STROBE recommendations for quality assurance.

## Results

Table 1 reports the main demographic and clinical characteristics of the 143 enrolled patients. Of note, the majority of patients were aged ≥ 65 years, displayed a high multimorbidity burden, as measured by the CIRS, and roughly one third of them was frail, as measured by the EFS.

**Table 1:** Main demographic and clinical characteristics of the cohort of patients

Total number of patients, n	143
Age (years), median (IQR)	69 (52–79)
Age groups (years), n (%)	
18–64	60 (42.0)
≥ 65	73 (58.0)
Sex, n (%)	
M	69 (48.3)
F	74 (51.7)
CIRS comorbidity index, median (IQR)	3 (2–4)
CIRS severity index, median (IQR)	1.6 (1.4–1.8)
CIRS comorbidity index ≥ 3, n (%)	
No	86 (60.1)
Yes	57 (39.9)
BMI class, n (%)	
< 18.5	15 (10.5)
18.5–24.9	71(49.6)
25–29.9	34 (23.8)
≥ 30	23 (16.1)
Admission diagnosis, n (%)	
Circulatory ICD9 chapter	30 (21)
Respiratory ICD9 chapter	22 (15)
Gastroenteric ICD9 chapter	18 (13)
Symptoms ICD9 chapter	43 (30)
Others	30 (21)
Resilience, mean (SD)	60.6 (19.1)
Edmonton > 5, n (%)	
No	94 (66.2)
Yes	49 (33.8)
Barthel index, mean (SD)	92.5 (12.7)
Income < 1000 €/mon, n (%)	
No	85 (59.4)
Yes	58 (40.6)
Living alone, n (%)	
No	107 (74.8)
Yes	36 (25.2)
Schooling ≤ 8, n (%)	
No	102 (71.3)
Yes	41 (28.7)
SBT ≥ 9, n (%)	
No	113 (79.6)
Yes	29 (20.4)

*Abbreviations*: *CIRS* Cumulative Illness Rating scale, *ICD9* International Classification of Diseases 9.^th^ revision, *IQR* Interquartile range, *SD* Standard deviation

In Table 2 we reported the demographic and clinical variables in relation to resilience as a continuous variable. Resilience was significantly lower in frail (*p* = 0.010), elderly patients (*p* = 0.021), in those lacking functional autonomy (*p* = 0.032), in those with a higher CIRS severity index (*p* = 0.028) and in more cognitively impaired patients (*p* = 0.028), and in those with a low educational level (*p* = 0.032). No relation between resilience and sex, BMI, admission diagnosis, income, living alone, and LOS (Spearman's rho -0.0445; *p* = 0.597) was found.

**Table 2:** Demographic and clinical variables in relation to resilience as a continuous variable (generalised linear regression model, univariable analysis)

	N (%)	Mean ± SD	Difference	95% CI	*p*-value
**Edmonton frail scale and resilience**					0.010
Edmonton ≤ 5	94 (65.7)	63.41 ± 17.96	0 (base)		
Edmonton > 5	49 (34.3)	54.69 ± 20.38	-8.73	-15.32 to -2.13	
**Sex and resilience**					0.652
M	69 (48.3)	61.32 ± 18.48	0 (base)		
F	74 (51.7)	59.86 ± 19.88	-1.45	-7.81 to 4.90	
**Age group and resilience**					0.021
18–64	60 (42.0)	66.25 ± 18.41	0 (base)		
≥ 65	73 (58.0)	56.91 ± 18.21	-9.34	-17.32 to -0.78	
**BMI class and resilience**					0.785
< 18.5	15 (10.5)	60.47 ± 19.93	0 (base)		
18.5–24.9	71 (49.6)	62.15 ± 18.58	1.69	-9.15 to 12.53	0.759
25–29.9	34 (23.8)	58.59 ± 20.54	-1.88	-13.70 to 9.95	0.754
≥ 30	23 (16.1)	58.65 ± 19.22	-1.81	-14.48 to 10.85	0.777
**Admission diagnoses**					
^#^Circulatory ICD9 chapter	30 (21.0)	56.63 ± 18.68	0 (base)		
^#^Respiratory ICD9 chapter	22 (15.4)	61.73 ± 20.71	5.09	-5.60 to 15.79	0.348
^#^Gastroenteric ICD9 chapter	18 (12.6)	64.83 ± 17.20	8.20	-3.16 to 19.56	0.156
^#^Symptoms ICD9 chapter	43 (30.0)	61.53 ± 19.49	4.90	-4.16 to 13.97	0.287
^#^Other ICD9 chapters	30 (21.0)	59.70 ± 19.62	3.07	-6.77 to 12.91	0.539
**Barthel index**					0.032
Barthel ≥ 60	136 (95.1)	61.35 ± 18.66	0 (base)		
Barthel < 60	7 (4.9)	45.43 ± 24.01	-15.92	-30.41 to -1.42	
**CIRS; CIRS severity and resilience**					0.028
CIRS < 3	86 (60.1)	63.42 ± 17.74	0 (base)		
CIRS ≥ 3	57 (39.9)	56.26 ± 20.55	-7.16	-13.54 to -0.77	
**Income and resilience**					0.073
Income ≥ 1000 €	85 (59.4)	62.94 ± 18.84	0 (base)		
Income < 1000 €	58 (40.6)	57.09 ± 19.27	-5.85	-12.26 to 0.55	
**Living alone and resilience**					0.519
No	107 (74.9)	61.17 ± 18.88	0 (base)		
Yes	36 (25.1)	58.78 ± 20.16	-2.39	-9.71 to 4.92	
**Schooling and resilience**					0.032
Schooling > 8 years	102 (71.3)	62.75 ± 19.02	0 (base)		
Schooling ≤ 8 years	41 (28.7)	55.15 ± 18.65	-7.60	-14.51 to -0.68	
**Short blessed test and resilience**					0.028
SBT < 9	113 (79.0)	62.68 ± 18.94	0 (base)		
SBT ≥ 9	30 (21.0)	54.17 ± 16.28	-8.51	-16.10 to -0.92	

*Abbreviations*: *BMI* Body Mass Index, *CI* Confidence interval, *CIRS* Cumulative Illness Rating Scale, *SBT* Short blessed Test, *SD* Standard deviation

Demographic and clinical variables in relation to frailty are reported in Supplementary Table 1. Similarly to what found for resilience, also frail patients (EFS > 5) were significantly older (*p* < 0.001), had a greater disease burden as measured by CIRS comorbidity (*p* < 0.001) and severity indexes (*p* < 0.001), were more dependent (*p* < 0.001), more cognitively impaired (*p* < 0.001), and displayed a lower educational level (*p* = 0.011) compared to non-frail patients. Notably, BMI, LOS, lower household income and living alone were not statistically different between frail and non-frail patients (data not shown in Table).

In a multivariable analysis for factors affecting resilience according to different age groups (Table 3), after the exclusion of variables which turned out to be collinear, we found that frailty as measured by the EFS (*p* = 0.022) and dependency according to the Barthel index (*p* = 0.031) were the only statistically significant factors related to lower resilience in the age groups 18–64 and ≥ 65 years, respectively. Although only a minority of patients had a Barthel index < 60, we could not use this variable as continuous due to a relevant ceiling effect (i.e., the first tertile and the first quartile cut-offs were 88 and 95, respectively). Given that at univariable analysis there was no association of admission diagnosis and resilience, this variable was not included in the multivariable analysis.

**Table 3:** Multivariable sub-group analysis (generalised linear regression model) for factors affecting resilience according to age groups (18–64 and ≥ 65 years)

	**Age 18–64**	*(model p-value 0.078)*		**Age ≥ 65**	*(model p-value 0.240)*	
	Adjusted Difference	95% CI	*p*-value	Adjusted Difference	95% CI	*p*-value
**Edmonton**			0.022			0.817
Edmonton < 5	0 (base)			0 (base)		
Edmonton > 5	-14.6	-27.14 to -2.17		1.2	-8.98 to 11.35	
**Barthel index**			0.192			0.031
Barthel > 60	0 (base)			0 (base)		
Barthel < 60	25.5	-13.18 to 64.11		-17.5	-33.37 to -1.63	
**Short blessed test**			0.800			0.664
SBT < 9	0 (base)			0 (base)		
SBT > 9	-3.10	-27.49 to 21.30		-2.2	-12.02 to 7.71	
**Income**			0.175			0.487
Income > 1000 €	0 (base)			0 (base)		
Income < 1000 €	-6.7	-16.36 to 3.05		-3.1	-11.86 to 5.70	
**CIRS; CIRS severity**			0.967			0.316
CIRS < 3	0 (base)			0 (base)		
CIRS > 3	0.26	-12.05 to 12.56		-4.77	-14.20 to 4.65	
**Schooling**			0.341			0.685
Schooling > 8 years	0 (base)			0 (base)		
Schooling < 8 years	-11.7	-36.28 to 12.78		1.8	53.71 to 68.62	

*Abbreviations*: *CI* Confidence interval, *CIRS* Cumulative Illness Rating Scale, *SBT* Short blessed Test

## Discussion

We herein found that, in an internal medicine setting comprising of a majority of elderly patients with a great disease burden, resilience and frailty were inversely correlated and both associated with ageing, dependency, cognitive impairment, and low educational level. These correlations seem to be a feature of this setting as a whole, as they turned out not to be dependent on the admission diagnosis. The fact that, in our study, the mean resilience level (60.6) turned out to be rather low when compared to other available settings, such as that of geriatric outpatients (66.2) [32], corroborates the importance of the clinical context. As we mentioned, resilience was lower in frail, elderly, dependent, multimorbid patients, in whom it has been poorly addressed in the past [33], as well as in those with a low educational level. Of note, at multivariable analysis, resilience was significantly and inversely related to frailty in adults (aged 18–64 years), and to dependency in older patients (≥ 65 years). We hypothesise that this may reflect the “natural history” of frailty which initially compromises main organ system functions and subsequently determines dependency, which both may severely affect resilience. Indeed, dependency is often preceded by a state of reduced capacity to respond to stressors due to a decline in functional reserves, i.e., frailty [34]. At any rate, we should highlight that our study was not designed for testing the directionality of these observed correlations, the explanation of which may be provided by future, ad hoc, longitudinal studies. Additionally, it should be noted that only a minority of patients in our study fell into the category of a Barthel index < 60 (seven cases), and this variable could not be used as continuous due to the substantial ceiling effect.

Consistently with previous observations, we found that also in an internal medicine setting advanced age [15, 35], multimorbidity [36], dependency [37], severe cognitive impairment [38], and low level of schooling [39, 40] were all associated with frailty. Of note, household income did not differ between frail and non-frail patients, and this might be explained by the universal public healthcare coverage which, at least partially, reduces socioeconomic differences in Italy. Additionally, the LOS did not differ between frail and non-frail patients, due to the allegedly higher in-hospital mortality in the former [16, 41, 42].

The results of the present study seem to point at the need for early detection of both low resilience and frailty, so to plan and prescribe ad hoc interventions for improving patients’ health. Since both resilience and frailty can be improved [43–46], corrective actions may potentially constitute a therapeutic advantage. For example, physical activity and nutritional interventions help improving both physical and mental functioning and are key for preventing frailty [19, 47, 48]. Resilience can be implemented through other interventions, including cognitive behavioural therapy [11] and, of note, according to a recent systematic review, resilience-promoting interventions have been attempted in twelve studies involving patients living with chronic conditions, including cancer, cardiovascular diseases, and diabetes [49]. Although the heterogeneity of the included studies partly affects the strength and generalisability of the findings, resilience enhancement programmes turned out to be of benefit on depression, anxiety, and quality of life, helping patients to recognise the changes in their lives and to improve adherence to the treatment plans [49].

To summarise, we have here provided further evidence about the necessity of integrating in clinical medicine the evaluation of resilience and frailty as it has been recently proposed by Hale and colleagues [50]. In this conceptual paper, the authors postulate that frailty can be considered as a phenotype of patients who lost resilience, and both factors may be potentially improved by taking actions on several other external or environmental determinants of health, such as the socioeconomic status and the level of education, this latter being correlated to both resilience and frailty in our study. The clinician has a central and active role in this process, being involved into the recognition of more vulnerable patients who may benefit from tailored interventions based on their specific needs.

Our study has some limitations that should be mentioned. The sample size is rather small, and it should be kept in mind that resilience can only be assessed in patients without severe cognitive impairment. Also, only a minority of patients displayed a low Barthel index, and hence this datum should not be overinterpreted. Instead, the strengths of our study include its prospective nature, which was not based on mere administrative data, and the fact that all data have been collected by the same research nurse and physicians who had been specifically trained before study initiation [25], and this ensures the homogeneity of the data. Our results should be interpreted in the light of this specific setting, i.e., that of hospitalised patients in an internal medicine ward, in which most patients are elderly and burdened by multimorbidity.

## Conclusions

To conclude, resilience and frailty turned out to be inversely related in adult hospitalised patients, while resilience and dependency were inversely related in a more advanced age. Whether frailty occurs before or after the occurrence of low resilience needs to be elucidated. Studies assessing the impact of these findings on important health outcomes are warranted, especially by including more compromised and dependent patients.

## Supplementary Information

**Additional file1: Supplementary Table 1.** Demographic and clinical variables in relation to frailty (as measured by the Edmonton Frail Scale).

## Data Availability

The datasets used and/or analysed during the current study are available from the corresponding author on reasonable request.

## Abbreviations

CBT: Cognitive behavioural therapy
CD-RISC: Connor-Davidson resilience scale
EFS: Edmonton Frail Scale

## References

[1] Windle G (2011). What is resilience? a review and concept analysis. Rev Clin Gerontol.

[2] Ungar M, Theron L (2020). Resilience and mental health: how multisystemic processes contribute to positive outcomes. Lancet Psychiatry.

[3] Connor KM, Davidson JRT (2003). Development of a new resilience scale: the Connor-Davidson Resilience Scale (CD-RISC). Depress Anxiety.

[4] Echezarraga A, Calvete E, González-Pinto AM, Las HC (2018). Resilience dimensions and mental health outcomes in bipolar disorder in a follow-up study. Stress Health.

[5] Adamu A, Mchunu G, Naidoo JR (2019). Stress and resilience among women living with HIV in Nigeria. Afr J Prim Health Care Fam Med.

[6] McGowan JA, Brown J, Lampe FC, Lipman M, Smith C, Rodger A (2018). Resilience and physical and mental well-being in adults with and without HIV. AIDS Behav.

[7] Zhang H, Zhao Q, Cao P, Ren G (2017). Resilience and quality of life: exploring the mediator role of social support in patients with breast cancer. Med Sci Monit.

[8] Udumyan R, Montgomery S, Fang F, Valdimarsdottir U, Fall K (2019). Stress resilience in late adolescence and survival among cancer patients: a Swedish register-based cohort study. Cancer Epidemiol Biomarkers Prev.

[9] Li X, Chen S, Zhang J, Li L, Li Y, Ye M (2021). Resilience process and its protective factors in long-term survivors after lung cancer surgery: a qualitative study. Support Care Cancer.

[10] Lenti MV, Cococcia S, Ghorayeb J, Di Sabatino A, Selinger CP (2020). Stigmatisation and resilience in inflammatory bowel disease. Intern Emerg Med.

[11] Joyce S, Shand F, Tighe J, Laurent SJ, Bryant RA, Harvey SB (2018). Road to resilience: a systematic review and meta-analysis of resilience training programmes and interventions. BMJ Open.

[12] Ratajová K, Blatný J, Poláčková Šolcová I (2020). Social support and resilience in persons with severe haemophilia: An interpretative phenomenological analysis. Haemophilia.

[13] Gmuca S, Xiao R, Urquhart A (2019). The role of patient and parental resilience in adolescents with chronic musculoskeletal pain. J Pediatr.

[14] McAllister MSJ, Vincent DA, Hassett DAL (2015). Psychological resilience, affective mechanisms and symptom burden in a tertiary-care sample of patients with fibromyalgia. Stress Health.

[15] Clegg A, Young J, Iliffe S, Rikkert MO, Rockwood K. Frailty in elderly people. Lancet. 2013;381:752–62. 10.1016/S0140-6736(12)62167-9 PMC4098658 23395245

[16] Hoogendijk EO, Afilalo J, Ensrud KE, Kowal P, Onder G, Fried LP (2019). Frailty: implications for clinical practice and public health. Lancet.

[17] Hajek A, Brettschneider C, Posselt T (2016). Predictors of frailty in old age - results of a longitudinal study. J Nutr Health Aging.

[18] Tarazona-Santabalbina FJ, Gómez-Cabrera MC, Pérez-Ros P (2016). A multicomponent exercise intervention that reverses frailty and improves cognition, emotion, and social networking in the community-dwelling frail elderly: a randomized clinical trial. J Am Med Dir Assoc.

[19] Abizanda P, López MD, García VP (2015). Effects of an oral nutritional supplementation plus physical exercise intervention on the physical function, nutritional status, and quality of life in frail institutionalized older adults: the activnes study. J Am Med Dir Assoc.

[20] Trendelenburg AU, Scheuren AC, Potter P, Müller R, Bellantuono I (2019). Geroprotectors: A role in the treatment of frailty. Mech Ageing Dev.

[21] Woods NF, LaCroix AZ, Gray SL (2005). Frailty: Emergence and consequences in women aged 65 and older in the Women’s Health Initiative observational study. J Am Geriatr Soc.

[22] Rolfson DB, Majumdar SR, Tsuyuki RT, Tahir A, Rockwood K (2006). Validity and reliability of the Edmonton Frail Scale. Age Ageing.

[23] Corazza GR, Klersy C, Formagnana P, Lenti MV, Padula D (2017). Consensus Panel. A consensus for the development of a vector model to assess clinical complexity. Intern Emerg Med..

[24] Lenti MV, Klersy C, Brera AS (2020). Clinical complexity and hospital admissions in the December holiday period. PLoS ONE.

[25] Lenti MV, Klersy C, Brera AS, Benedetti I, Ciola M, Bertolino G, Corazza GR (2019). Reproducibility in the assessment of the components of a clinical complexity index. J Gen Intern Med.

[26] Connor, KM, Davidson JR. Translations of the CD-RISC. Connor-Davidson Resilience Scale. Available at: http://www.connordavidson-resiliencescale.com/translations.php 10.1002/da.10113 12964174

[27] He Y, Li LW, Hao Y (2020). Assessment of predictive validity and feasibility of Edmonton Frail Scale in identifying postoperative complications among elderly patients: a prospective observational study. Sci Rep.

[28] Linn BS, Linn MW, Gurel L (1968). Cumulative illness rating scale. J Am Geriatr Soc.

[29] Mahoney FI, Barthel DW (1965). Functional evaluation: The Barthel Index. Md State Med J.

[30] Katzman R, Brown T, Fuld P, Peck A, Schechter R, Schimmel H (1983). Validation of a short Orientation-Memory-Concentration Test of cognitive impairment. Am J Psychiatry.

[31] Shah S, Vanclay F, Cooper B (1989). Improving the sensitivity of the Barthel Index for stroke rehabilitation. J Clin Epidemiol.

[32] Callegari C, Bertù L, Caselli I (2016). Resilience in older adults: influence of the admission in nursing home and psychopathology. Neuropsychiatry (London).

[33] Ong BN, Richardson JC, Porter T, Grime J (2014). Exploring the relationship between multi-morbidity, resilience and social connectedness across the lifecourse. Health (London).

[34] Rodriguez-Mañas L, Fried LP (2015). Frailty in the clinical scenario. Lancet.

[35] Wleklik M, Uchmanowicz I, Jankowska EA (2020). Multidimensional approach to frailty. Front Psychol.

[36] Boeckxstaens P, Vaes B, Legrand D, Dalleur O, De Sutter A, Degryse JM (2015). The relationship of multimorbidity with disability and frailty in the oldest patients: a cross-sectional analysis of three measures of multimorbidity in the BELFRAIL cohort. Eur J Gen Pract.

[37] At J, Bryce R, Prina M (2015). Frailty and the prediction of dependence and mortality in low- and middle-income countries: a 10/66 population-based cohort study. BMC Med.

[38] Kulmala J, Nykänen I, Mänty M, Hartikainen S (2014). Association between frailty and dementia: a population-based study. Gerontology.

[39] Hoogendijk EO, van Hout HP, Heymans MW (2014). Explaining the association between educational level and frailty in older adults: results from a 13-year longitudinal study in the Netherlands. Ann Epidemiol.

[40] Rivas-Ruiz F, Machón M, Contreras-Fernández E (2019). Group GIFEA. Prevalence of frailty among community-dwelling elderly persons in Spain and factors associated with it. Eur J Gen Pract..

[41] Mousa A, Savva GM, Mitnitski A (2018). Is frailty a stable predictor of mortality across time? Evidence from the cognitive function and ageing studies. Age Ageing.

[42] Hogan DB, Freiheit EA, Strain LA (2012). Comparing frailty measures in their ability to predict adverse outcome among older residents of assisted living. BMC Geriatr.

[43] Fiatarone MA, O'Neill EF, Ryan ND (1994). Exercise training and nutritional supplementation for physical frailty in very elderly people. N Engl J Med.

[44] Lekan DA (2018). Aging, frailty, and resilience. J Psychosoc Nurs Ment Health Serv.

[45] Padesky CA, Mooney KA (2012). Strengths-based cognitive-behavioural therapy: a four-step model to build resilience. Clin Psychol Psychother.

[46] Petriwskyj A, Parker D, O'Dwyer S, Moyle W, Nucifora N (2016). Interventions to build resilience in family caregivers of people living with dementia: a comprehensive systematic review. JBI Database System Rev Implement Rep.

[47] McPhee JS, French DP, Jackson D, Nazroo J, Pendleton N, Degens H (2016). Physical activity in older age: perspectives for healthy ageing and frailty. Biogerontology.

[48] Kidd T, Mold F, Jones C (2019). What are the most effective interventions to improve physical performance in pre-frail and frail adults? A systematic review of randomised control trials. BMC Geriatr.

[49] Kim GM, Lim JY, Kim EJ, Park SM (2019). Resilience of patients with chronic diseases: A systematic review. Health Soc Care Community.

[50] Hale M, Shah S, Clegg A (2019). Frailty, inequality and resilience. Clin Med (Lond).

