# Supplementary material for: Resilience is associated with frailty and older age in hospitalised patients
Source: BMC Geriatr. 2022 Jul 10;22:569. doi: 10.1186/s12877-022-03251-9 (PMC9275243; doi:10.1186/s12877-022-03251-9)
Supplement: Supplementary file 1 — Additional file1: Supplementary Table 1. Demographic and clinical variables in relation to frailty (as measured by the Edmonton Frail Scale). [file 12877_2022_3251_MOESM1_ESM.docx]

**Supplementary Table 1**. Demographic and clinical variables in relation to frailty (as measured by the Edmonton Frail Scale).

| **Variable** | ***Non-frail (n=94)** | ***Frail (n=49)** | **p-value** |
| --- | --- | --- | --- |
| Age (years) | 54.1±19.1 | 73.2±14.2;69.10-77.36 | <0.001 |
| Age ≥65 years, n (%) | 45 (48) | 37 (77) | 0.001 |
| Female, n (%) | 49 (52) | 25 (52) | 1.00 |
| Resilience (mean±SD) | 63.4±18.0 | 54.7±20.4 | 0.001 |
| CIRS comorbidity index (mean±SD) | 2.63±1.31 | 4.38±1.88 | <0.001 |
| CIRS severity index (mean±SD) | 1.52±0.24 | 1.88±0.34 | <0.001 |
| LOS (days), median (IQR) | 11 (8-18) | 15 (10-19) | 0.129 |
| BMI (mean±SD) | 24.6±5.0 | 25.5±6.6 | 0.34 |
| ^#^Circulatory ICD9 chapter, n (%) | 15 (50) | 15 (50) | NA |
| ^#^Respiratory ICD9 chapter, n (%) | 13 (59.1) | 9 (40.9) | NA |
| ^#^Gastroenteric ICD9 chapter, n (%) | 12 (66.7) | 6 (33.3) | NA |
| ^#^Symptoms ICD9 chapter, n (%) | 32 (76.2) | 10 (23.8) | NA |
| ^#^Other ICD9 chapters, n (%) | 22 (73.3) | 8 (26.7) | NA |
| Barthel index (mean±SD) | 97.0±5.4 | 83.6±17.6 | <0.001 |
| Short blessed test, median (IQR) | 0 (0-4) | 6 (1-12) | <0.001 |
| Schooling ≤8 years, n (%) | 20 (21) | 21 (44) | 0.006 |

*According to the Edmonton Frail Scale, a score of ≤5 identifies non-frail patients, while a score of >5 identifies frail patients. ^#^These are the main admission diagnoses. The p-value (Fisher’s exact) for the whole admission diagnoses is 0.166. Abbreviations: BMI, Body Mass Index; CI, confidence interval; CIRS, Cumulative Illness Rating Scale; ICD9, International Classification of Diseases 9^th^ revision; LOS, length of stay, NA, not assessed.
